# Standardized phytotherapic extracts rescue anomalous locomotion and electrophysiological responses of TDP-43 *Drosophila melanogaster* model of ALS

**DOI:** 10.1038/s41598-018-34452-1

**Published:** 2018-10-30

**Authors:** Riccardo Maccioni, Maria Dolores Setzu, Giuseppe Talani, Paolo Solari, Ameya Kasture, Sonja Sucic, Simona Porru, Patrizia Muroni, Enrico Sanna, Sanjay Kasture, Elio Acquas, Anna Liscia

**Affiliations:** 1 University of Cagliari, Department of Biomedical Sciences, Monserrato, 09042 Italy; 2 National Research Council (CNR), Institute of Neuroscience, Monserrato, 09042 Italy; 3 Medical University of Vienna, Institute of Pharmacology, Center of Physiology and Pharmacology, Vienna, A-1090 Austria; 4 University of Cagliari, Department of Life and Environmental Sciences, Cagliari, 09126 Italy; 5 Pinnacle Biomedical Research Institute, Bhopal, 462003 India

## Abstract

Findings from studies using animal models expressing amyotrophic lateral sclerosis (ALS) mutations in RNA-binding proteins, such as Transactive Response DNA-binding protein-43 (TDP-43), indicate that this protein, which is involved in multiple functions, including transcriptional regulation and pre-mRNA splicing, represents a key candidate in ALS development. This study focuses on characterizing, in a *Drosophila* genetic model of ALS (TDP-43), the effects of *Mucuna pruriens* (*Mpe*) and *Withania somnifera* (*Wse*). Electrophysiological and behavioural data in TDP-43 mutant flies revealed anomalous locomotion (i.e. impaired climbing with unexpected hyperactivity) and sleep dysregulation. These features, in agreement with previous findings with a different ALS model, were at least partially, rescued by treatment with *Mpe* and *Wse*. In addition, electrophysiological recordings from dorsal longitudinal muscle fibers and behavioral observations of TDP-43 flies exposed to the volatile anaesthetics, diethyl ether or chloroform, showed paradoxical responses, which were normalized upon *Mpe* or *Wse* treatment. Hence, given the involvement of some potassium channels in the effects of anaesthetics, our results also hint toward a possible dysregulation of some potassium channels in the ALS-TDP-43 *Drosophila* model, that might shed new light on future therapeutic strategies pertaining to ALS.

## Introduction

Alterations in the expression levels of the highly conserved transactive response DNA binding protein 43 (TDP-43), cause amyotrophic lateral sclerosis (ALS) and fronto-temporal dementia (FTD)^1–5^. Apart from mutations in the genes encoding superoxide dismutase-1 (SOD1) and fused in sarcoma (FUS), variants of TDP-43 have been reported in almost all the patients afflicted with ALS^6,7^. TDP-43 is an ubiquitously expressed nuclear protein, harboring two RNA recognition motifs: a nuclear localization sequence with a nuclear export signal and a glycine-rich C-terminus^1^. Along with regulating transcriptional repression and splicing, TPD-43 also modulates micro RNA biogenesis^8–13^.

Both, the over-expression of TDP-43, leading to its cytoplasmic accumulation (*i*.*e*. cytotoxic gain-of-function), and the nuclear-loss of TDP-43 (*i*.*e*. loss of function), have been shown to progress to neurodegeneration (reviewed in^9,14^). A number of studies performed in mice, zebrafish and *Drosophila* have shown that both gain- and loss- of TDP-43 function lead to impaired motor behaviour and loss of motoneurons^15–19^. Interestingly, in Zebra fish and *Drosophila*, the loss-of-function phenotype associated with motor impairment could be rescued by expressing wild-type TDP-43, suggestive of plasticity in the neural circuit^17,18^. Moreover, as suggested by Cragnaz *et al*.^20^, the age-related drop in TDP-43 expression might likely be a dramatic event which promotes the onset of the human disease.

Remarkably, albeit Riluzole has been approved more than 20 years ago by FDA and considered the recent approval of Radicava (edaravone) to treat patients with ALS, the most part of the currently available treatment still remains somewhat of a conundrum, making the pursuit for new therapeutic strategies essential.

Several traditional medicinal systems, such as Ayurveda, provide a thorough body of literature on medicinal plants, which can be exploited by rational drug design and development of novel pharmaceutical approaches. *Mucuna pruriens* (L.) DC. (*Mpe*) and *Withania somnifera* (L.) Dunal *(Wse)* have been widely used in Ayurvedic medicine for their potential effects in treating neurodegenerative disorders^21^. Although isolated components of *Wse* have already been studied in detail, pointing out withanolides and withanone for their crucial role in neuroprotection^22–24^, in our previous studies we tested the effectiveness of methanolic extracts, of *Mpe* and *Wse* in *Drosophila* models of Parkinson’s disease^25,26^, and of ALS (ALS-hSOD1^27^). In the hSOD1 model, treatment with *Mpe* and *Wse* remedied the locomotor defects present in this fly phenotype^27^. Based on the above observations and according to the study by Cragnaz *et al*.^20^ and Feiguin *et al*.^28^ that correlated TDP-43 levels with motor impairment, we postulated that *Mpe* and *Wse* treatments may prove effective in restoring the function of motoneurons in a partial TDP-43-loss-of-function *Drosophila* model. Our present study exploits advanced genetic tools in *Drosophila*, to mimic a phenotype associated to reduced levels of TDP-43 expression in motoneurons and examine whether *Mpe* and *Wse* hold therapeutic potential in the treatment of ALS.

## Results

### *Mpe* or *Wse* treatment ameliorates the climbing and locomotion behaviour of TDP-43 mutant flies

**Figure 1:**
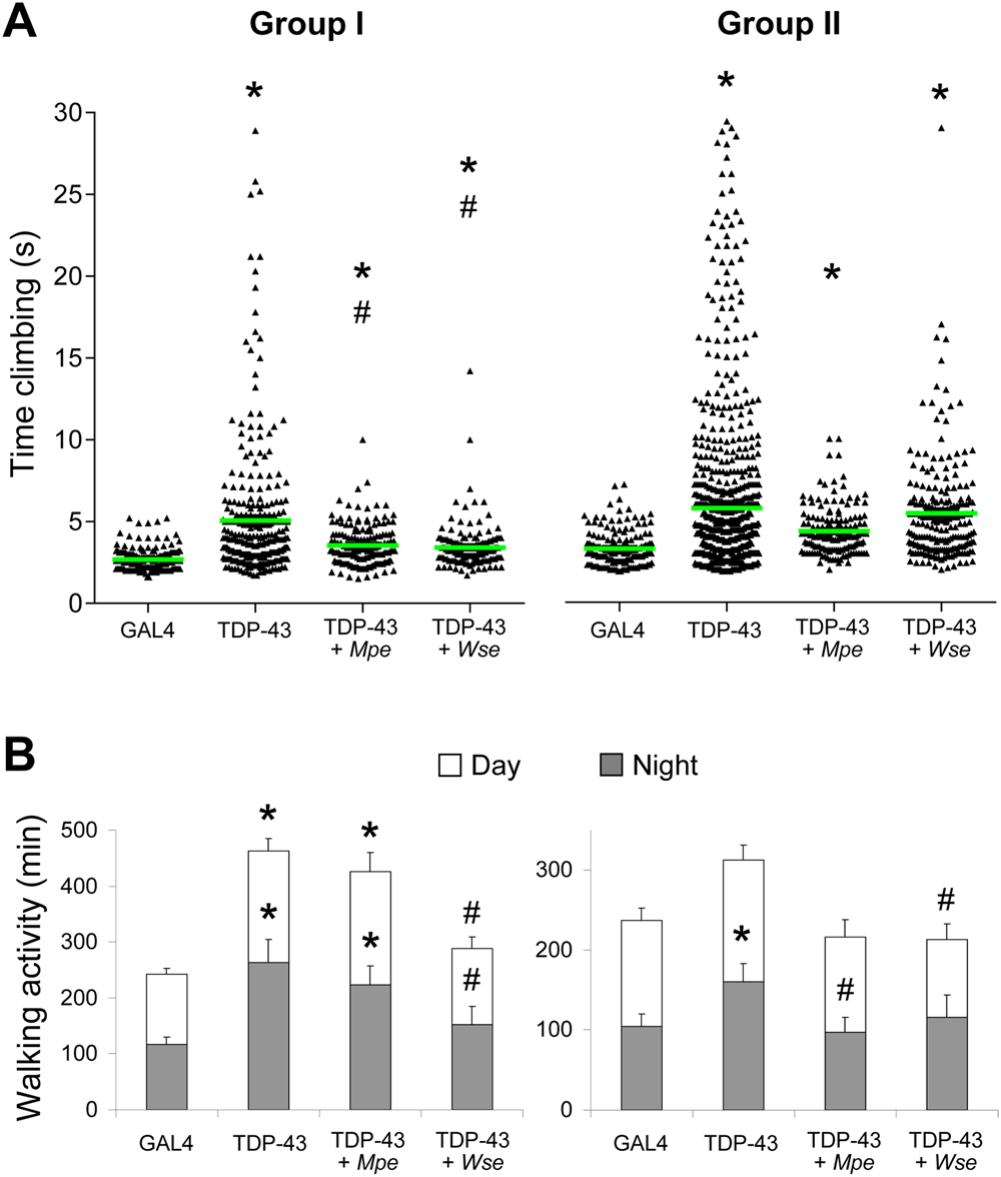
*Mpe* or *Wse* treatment ameliorates the climbing and locomotion behaviour of TDP-43 mutant flies. Effects of *Mucuna pruriens* (*Mpe*) or *Withania somnifera* (*Wse*) extracts on the climbing **(A)** and walking **(B)** activity of adult GAL4 flies, untreated mutants (TDP-43) and *Mpe-* or *Wse*-treated TDP-43. The treatment was administered during the adult stage and its effect was assayed in two age groups (Group I: 3–6 day old flies, Group II: 10–15 day old flies). In each set of data in (**A**) the green horizontal line indicates the mean of the corresponding distribution. * and ^#^ indicate p < 0.05, one-way ANOVA (non parametric) followed by Dunn’s multiple comparisons test, compared to GAL4 and TDP-43, respectively. The histograms in (**B**) display the walking activity during the 12-h daytime (white) and 12-h nighttime (black) periods. * and ^#^ indicate p < 0.05, one-way ANOVA followed by HSD post-hoc test, compared to GAL4 and TDP-43, respectively.

The GAL4-Upstream Activation Sequence (UAS) system was used to specifically knockdown TAR DNA-binding protein-43 homolog (TBPH) in motoneurons, in order to obtain a partial depletion of the protein. D42 GAL4 driven RNA interference (RNAi) mediated knockdown of TBPH (hence forth called as TDP-43 mutant flies) impaired the climbing and locomotion behaviour in flies (Fig. 1).

The behavioural performance of *TBPH* knockdown was studied in two age groups (I: 3–6 and II: 10–15 day-old male flies). As shown in Fig. 1A, TDP-43 mutant flies showed a significant increase in the climbing time in both age groups tested with respect to the control. We also assessed the effects of *Mpe* or *Wse* on the climbing behaviour of TDP-43 mutant flies, treated with *Mpe* or *Wse* supplemented in food (0.1% w/w) upon eclosion. Treatment of group I TDP-43 mutant flies with either *Mpe* or *Wse* (0.1% w/w) significantly reduced the climbing time (p < 0.05, ANOVA, non parametric), suggesting partial recovery of the motoneuron impairment. In the second group, no significant difference was found between treated and untreated TDP-43 mutant flies. Therefore, the climbing performance of TDP-43 mutant flies declines with age, both in treated and untreated ones, even if in the latter a trend to recovery was found.

After observing a recovery in the climbing behaviour of TDP-43-treated flies, we reasoned that the overall locomotion activity of TDP-43 mutant flies could be restored upon treatment (Fig. 1B). Hence, the locomotion of groups I and II flies was recorded using a drosophila activity monitor (DAM2, trikinetics) and plotted as daytime and nighttime activity. The group I and II TDP-43 mutant flies showed heightened locomotion activity compared to GAL4 control flies. *Mpe* treated (0.1% w/w) group I TDP-43 flies exhibited a slight reduction in the overall locomotion activity compared to TDP-43 flies. On the other hand, *Wse* (0.1% w/w) pre-treated group-I TDP-43 flies showed a markedly decreased daytime and nighttime locomotion activity. Interestingly, the overall locomotion activity of *Mpe* treated group II TDP-43 flies was comparable to that of GAL4 flies, indicating that continuous administration of *Mpe* may have beneficial effects. In addition, group II TDP-43 flies showed locomotion comparable to GAL4 flies after *Wse* treatment. Collectively, these findings suggest that *Mpe* and *Wse* ameliorate the hyperactive phenotype of TDP-43 flies.

### *Mpe or Wse* restore changes in ePSPs recorded from DLM of TDP-43 mutant flies

**Figure 2:**
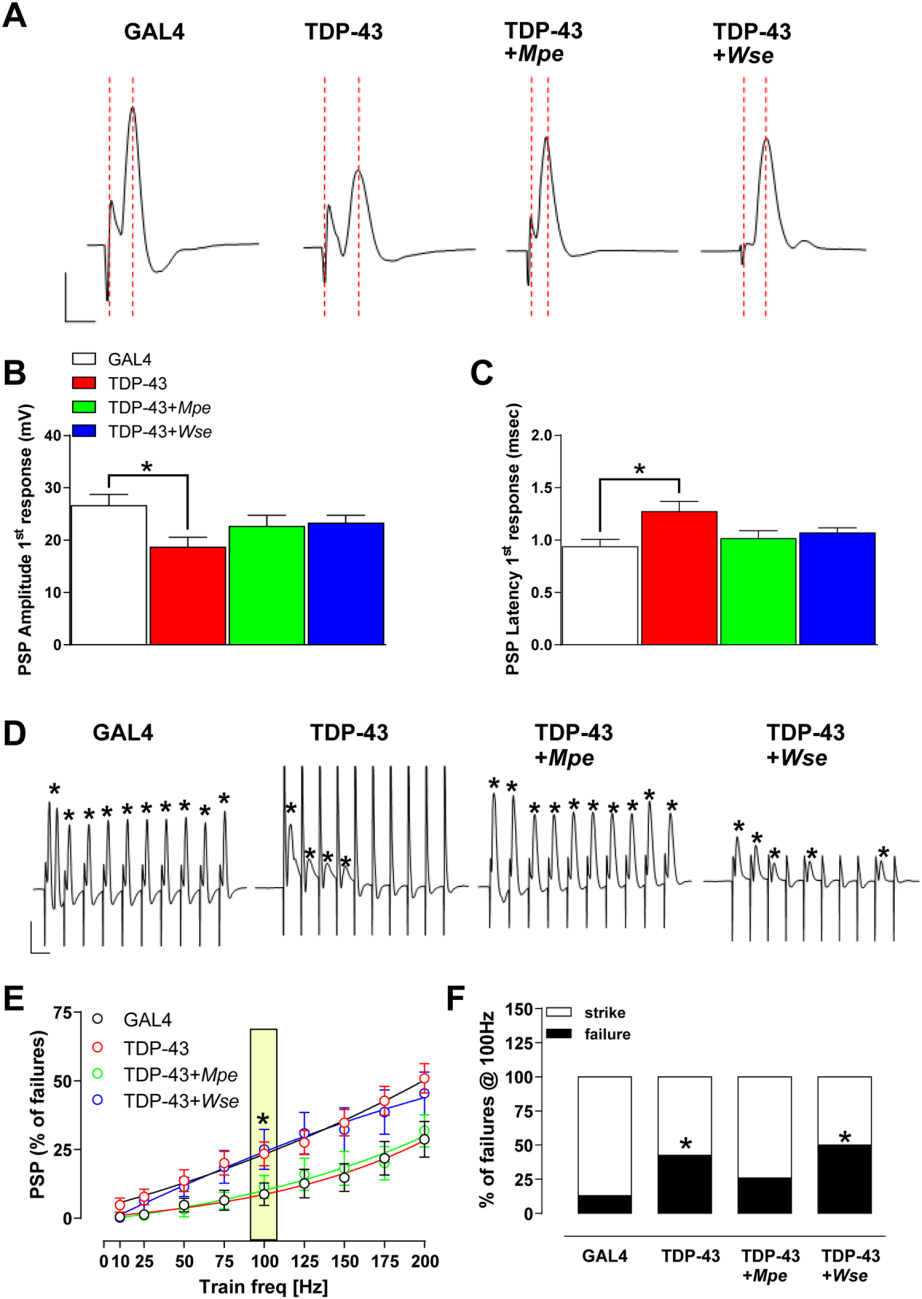
Effect of TDP-43 loss-of-function mutation and treatment with *Mpe* or *Wse* on electrophysiological parameters recorded from DLM. (**A**) Representative traces obtained from different experimental conditions, in which ePSP latency is calculated as the time (ms) from stimulus application to the peak of post-synaptic potential (PSP) (red scattered lines), and PSP peak is calculated by measuring the maximal amplitude of the response starting from the baseline. Scale bar, 10 mV/1 ms. (**B**) Ámplitude (mV) and (**C**) latency (ms) recorded in flies from different experimental groups. Bar graphs represent the mean  ± standard error of the mean (SEM) of ePSPs. * indicates p < 0.05 compared to GAL4, one-way ANOVA, followed by Bonferroni post-hoc test. N = 22 for all experimental groups. (**D**) Representative traces obtained from different experimental groups in which PSPs were evoked in response to 10 stimuli delivered at 100 Hz. Scale bar 10 mV/20 ms. * indicates the presence of the event. (**E**) Scatter plot graphs showing the changes in ePSPs amplitude following stimulation at increasing frequency (from 10 to 200 Hz) of a train of 10 consecutive stimuli (the effect at 100 Hz is highlighted in yellow). All values are expressed as the mean ± SEM of the % of failure observed in every train. * indicates p < 0.05 vs GAL4. (**F**) Bar graph representing the averaged % of failure that was plotted in all experimental groups. “Strike” is the % of recordings where failure was not greater that 10%, while “failure” is the number of recordings where the % of failure was greater than 20%. Data corresponds to the % of failures evaluated @ 100 Hz. * indicates p < 0.05 compared to GAL4, unpaired t-test.

We then examined the function of the dorsal longitudinal muscle (DLM) neuromuscular junction in TDP-43 mutant flies. Kinetic properties (i.e. the amplitude and latency) of evoked post-synaptic potentials (ePSPs), recorded from the DLM after Giant Fibre System (GFS) electrical stimulation, were evaluated under basal conditions. The amplitude and latency of ePSPs recorded from the DLM muscle of GAL4 flies had an averaged value of 26.13 ± 2.59 mV and 0.94 ± 0.07 ms, respectively (Fig. 2A–C).

Interestingly, ePSPs recorded in TDP-43 mutant flies were characterized by a significant decrease in amplitude (20.32 ± 2.7 mV; p < 0.05 vs GAL4, ANOVA) and an increase in latency (1.4 ± 1.1 ms; p < 0.05 vs GAL4, ANOVA) (Fig. 2A–C). These data suggest that the TDP-43 mutation may hamper the function of GFS-DLM neuronal conduction as well as muscle efficiency. As observed in previous reports with flies carrying different SOD1 mutations^27^, the treatment of TDP-43 mutants with *Mpe* or *Wse* significantly restored the changes in amplitude and latency of ePSPs, with values indistinguishable from those observed in GAL4 flies (p < 0.05; Fig. 2A–C).

### *Mpe* but not *Wse* ameliorates the ePSP response to high frequency stimulation of GFS in TDP-43 mutant flies

As reported previously^26,27,29^, recording the “frequency of following”, which consists of applying a train of 10 stimuli to the GFS at different frequencies (from 10 to 200 Hz, with steps of 25 Hz), allows physiological studies on the GFS-DLM muscle fibre. As expected, the number of failures in GAL4 flies was very low (8.7%) at 100 Hz but dramatically increased (28.6%) at 200 Hz (Fig. 2D,E). Remarkably, when TDP-43 flies were exposed to the same protocol, the rate of failures at both 100 and 200 Hz resulted significantly enhanced with respect to GAL4 flies (p < 0.05, ANOVA) (Fig. 2D–F). The treatment of TDP-43 flies with *Mpe* but not *Wse* was able to antagonize the increased failures found in TBP-43 flies in response to train stimulations (100 Hz), resulting in values comparable to those observed for the GAL4 flies (Fig. 2D–F).

### *Mpe* or *Wse* treatment modulates sleep parameters inTDP-43 mutant flies

**Figure 3:**
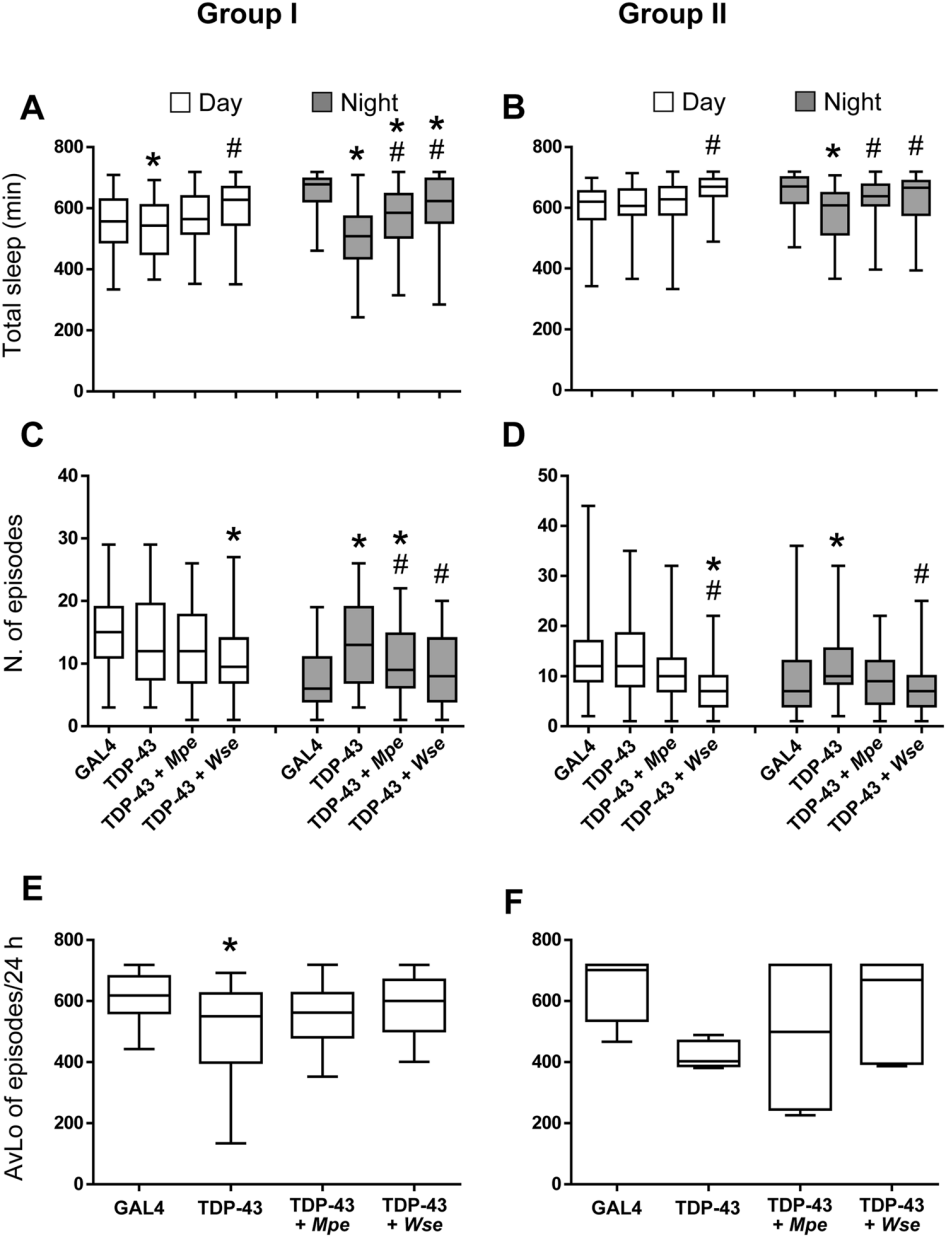
*Mpe* or *Wse* treatment modulates sleep parameters inTDP-43 mutant flies. Effects of *Mucuna pruriens* (*Mpe*) or *Withania somnifera* (*Wse*) extracts on total sleep (**A**,**B**), the number of sleep episodes (**C**,**D**) during the 12-h daytime (white) and 12-h nighttime (gray) periods and on the average of longest sleep episodes (AvLo)/24 h (**E**,**F**) of adult GAL4 flies, untreated mutants (TDP-43) and *Mpe-* and *Wse*-treated TDP-43 flies. The treatment was administered during the adult stage and its effect assessed in two groups (Group I: 7 days and Group II: 14 days) of the flies’ life-span. The top and bottom of the box and whisker plots show the upper and lower quartiles, respectively. The horizontal line in the middle indicates the median of the corresponding distribution, while the minimum and maximum observed values are indicated by the bars connected to the box. * and ^#^ indicate p < 0.05, one-way ANOVA followed by HSD post-hoc test, compared to GAL4 and TDP-43, respectively.

We next studied the effects of *Mpe* or *Wse* treatment (0.1% w/w) on sleep time of TDP-43 mutant flies. Both groups I and II TDP-43 mutant flies showed reduced nighttime sleep duration, compared to GAL4 control flies. In agreement with the locomotion data (Fig. 1B), TDP-43 flies of both age groups treated with *Mpe* or *Wse* showed a significantly restored the nighttime sleep duration (Fig. 3A,B).

Nighttime sleep is associated with long sleep episodes, which usually result in a reduced number of sleep episodes, a low number of long sleep episodes being indicative of a less fragmented sleep pattern^30^. TDP-43 mutant flies showed an increased number of sleep episodes compared to GAL4 flies in both age groups (Fig. 3C,D). *Mpe* treated group I and II TDP-43 mutant flies showed a reduced number of sleep episodes, although this reduction was not statistically significant for the flies of group II. *Wse* treatment significantly reduced the number of sleep episodes of TDP-43 mutant flies in both groups (Fig. 3C,D). The data are consistent with the notion that the TDP-43 mutation leads to sleep fragmentation, most likely due to hyperactivity^31^. The decrease in daily sleep (Fig. 3C,D) negatively correlated with the increase in locomotor activity. Moreover, the average of longest sleep episodes in TDP-43 mutants is significantly lower with respect to GAL4 and TDP-43 treated flies, thus suggesting that treatment ameliorates sleep fragmentation, although to a different extent (Fig. 3E,F).

### *Mpe* or *Wse* treatment prevents the effect of ether or chloroform administration on spontaneous PSPs in TDP-43 mutant flies

**Figure 4:**
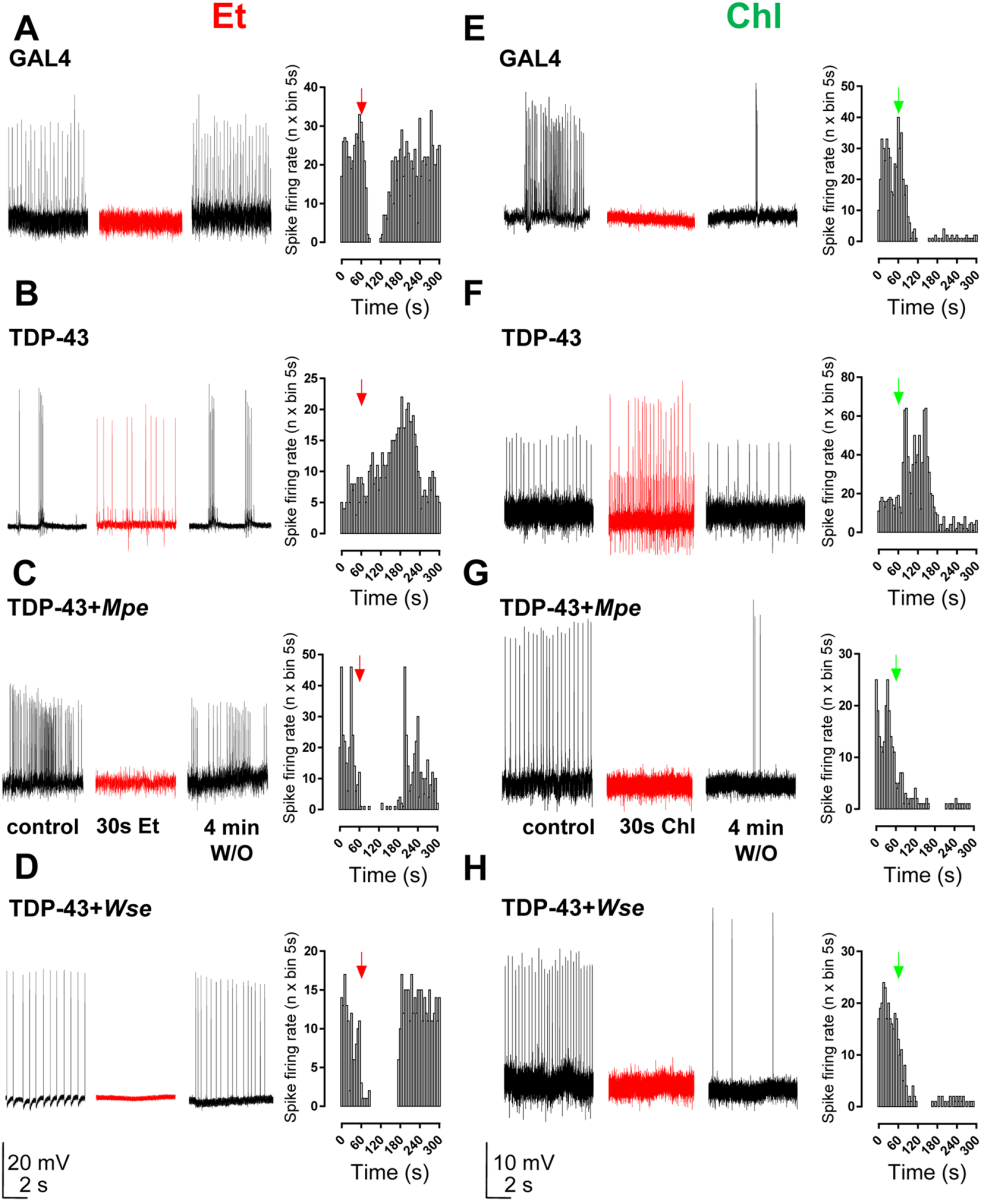
*Mpe* or *Wse* treatment prevents the effect of ether and chloroform administration on spontaneous PSPs in TDP-43 mutant flies. Treatment with *Mpe* or *Wse* rescues the effect of TDP-43 loss-of-function mutation to the enhancement of frequency of spike potentials due to *Drosophila* DLMs spontaneous contractions induced by 30 s of diethyl ether (Et) or chloroform (Chl) exposure. (**A**–**H**) Representative traces and plots obtained from GAL4 flies, TPD-43 treated and untreated mutants in which spontaneous PSPs were recorded in the absence, after 30 s and after 4 min wash-out of anaesthetic exposure. Arrows in graphs indicate when vapours of volatile anaesthetics delivery was begun. Scale bars: left panels, 20 mV/2 s, right panels 10 mV/2 s.

By considering that TDP-43 mutants show sleep parameters (fragmented sleep and motor activity) similar to those usually exhibited by Shaker flies (SH)^32^, in a separate set of these flies, we recorded the DLMs muscle contraction corresponding to a spontaneous wings beat, visualized as spike potentials frequency (Fig. 4), in the absence or after 30 s of diethyl ether (Et) or chloroform (Chl) vapour administration. For the sake of clarity, we specify that the noise level of traces was quite different between animals and strongly related to the experimental protocol by which, at variance with the experimental condition of data shown in Fig. 2, wings are left free to move, in order to also address the spontaneous activity of DLM.

Application of both volatile anaesthetics induced a rapid deep anaesthesia. Right after diethyl ether or chloroform application, the spontaneous activity of DLM was completely, but reversibly abolished (only for diethyl ether but not for chloroform) in GAL4 flies (Fig. 4A,E). Interestingly, when TDP-43 mutant flies were tested with the same protocol, the spike potentials frequency was markedly increased after 30 s of diethyl ether or chloroform application. In TDP-43 mutants treated with either *Mpe* or *Wse*, the effect of both anaesthetics application on spontaneous DLM contractions was similar to that observed in GAL4 flies (Fig. 4C–H). We tested the same protocol also in SH flies and we observed a dramatic increase of DLM contractions right after diethyl ether application (Supplementary Fig. S1), while chloroform administration failed to induce the same effect.

## Discussion

In our earlier studies in a *Drosophila* model of ALS-SOD1 overexpression, we reported that *Mpe* and *Wse* extracts ameliorated SOD1-induced pathologies^27^. We point out that our ALS TDP-43 mutants used in the present study differ with respect to the above cited genetic Dm model, being the current a model with a knock-down gene, that brings to a reduced level of TDP-43 expression. This leads to impaired climbing and locomotor abilities, although with a great inhomogeneity, that might be likely due to inhomogeneous levels of TDP-43, according to the previous study by Feiguin *et al*.^28^. Also, according to Cragnaz *et al*.^20^, age-related drop in TDP-43 expression may account for the worsening of climbing and locomotion activity in both untreated and treated mutants. In fact, scattering of data is dramatically increased in the oldest (second) group of flies.

Interestingly, also shorter sleep time and an increased number of nighttime sleep episodes were shown, effects that may be correlated with the fragmented sleep commonly observed in ALS patients^33^. The overall hyper locomotion of TDP-43 flies was recovered by *Wse* treatment, while *Mpe* treatment proved to be effective only in the 10–15 day old flies. In addition, treatment with *Mpe* and *Wse* improved the nighttime sleep duration and reduced the number of sleep episodes. The ePSPs recordings from DLM of TDP-43 mutant flies revealed that both *Mpe* and *Wse* may restore neuromotor function. In agreement with our results, a recent study on a mouse model of ALS, expressing the humanTDP-43^A315T^ mutation which affects motor function, showed that *Wse* treatment can reduce Nuclear Factor Kappa Beta (NF-κB) activation and neuroinflammation, and ameliorate motor defects^34^. An interesting possibility to interpret these results might be that *Mpe* and *Wse* optimize TDP-43 expression levels, as suggested also by Cragnaz *et al*.^20^ and Feiguin *et al*.^28^. Furthermore, TDP-43 mutant flies anaesthetized using both diethyl ether and chloroform (Supplementary Videos S1 and S2) showed a spastic paralysis of the legs coupled to a shaker movement not found in GAL4 flies (Supplementary Video S3); the latter is a phenotype typically seen in voltage-gated potassium channel mutant flies (Shaker and Ether-a’-Go-Go). The voltage-gated potassium channel mutant flies (SH), on the contrary, showed DLM contractions and shaker movements upon diethyl ether but not chloroform treatment (Supplementary Video S4). In mammals, diethyl ether and chloroform are known to activate TREK-1, a two-pore-domain potassium (K2P) channel^35^. K2P channels maintain hyperpolarized resting membrane potential of various cell types^36^. Our studies suggest that, similar to the mammalian TREK1 channel, at least one of the *Drosophila* K2P channels responds to diethyl ether and chloroform vapours and reasonably reduces glutamatergic transmission at the DLM motoneurons of wild-type flies^37^. In TDP-43 flies, impaired K2P functioning may fit with the disinhibition of presynaptic glutamate release, which eventually leads to increased spike potential frequency after diethyl ether and chloroform application (Fig. 4B). Importantly, the shaking TDP-43 phenotype was remedied in mutant flies treated with *Mpe* or *Wse* (see Supplementary Videos S5 and S6). Even if our findings on different effects on volatile anaesthetics suggest an involvement of K2P potassium channels, it would be important to deeply investigate this potential mechanism in TDP-43 mutant flies. Overall, the results reported in the present study suggest a novel and potential therapeutic approach toward the medication or at least the slow-down of the progression of ALS, and provide new evidences on the pathophysiology of this neurodegenerative disease suggesting future experiments to characterize the mechanism of this potential therapeutic action of *Mpe* and *Wse*.

All this notwithstanding, we acknowledge that this investigation refers to the effects of two extracts characterized by a complex composition and therefore we cannot make any hypothesis on the possible effects of single constituents that may possibly be responsible of the observed effects. Anyway, we cannot state that the effects of the individual constituents would reproduce the effects of the whole extract as exemplified by the case of *Hypericum perforatum* whose antidepressant effects cannot be reproduced by the individual active constituents^38^. In this respect the whole extracts of *Mucuna pruriens* and *Withania somnifera* were demonstrated to be effective in counteracting neurodegenerations as shown by us in previous studies^25–27^. Hence, at the present we can conclude that these whole extracts, besides being therapeutically effective, as shown in the present and other^22^ studies, are endowed with significant properties such as being safe^39–41^ as documented by the use in Ayurvedic medicine since many centuries, cheap and easy to be administered.” Obviously, it is necessary to assess the proper concentration and duration of treatment as these phytotherapic extracts exert their effects as a drug following a hormesis-like dose-response curve^27^.

## Methods

### Flies

Flies were reared on a standard cornmeal-yeast-agar medium in controlled environmental conditions (24–25 °C; 60% relative humidity; light/dark = 12/12). D42 GAL4 flies and RNAi line against *TBPH* were obtained from Bloomington Stock Center (*TBPH*-RNAi: 39014, D42GAL4: 42737). Flies were isogenized by backcrossing for more than five generations. Only male flies were used for the experiments.

### Pharmacological treatment

Flies were reared on a standard medium supplemented with *Mucuna pruriens* (*Mpe*) or *Withania somnifera* (*Wse*) extracts (kindly provided by Natural Remedies Pvt. Ltd., Bangalore, India). In order to follow good practice in publishing studies on herbal medicine^42^, we provided full details of the batches used (*Mpe* Batch No. RD/3208; *Wse* Batch No. PC/FWS1701003; Supplementary Figs S2 and S3).

In agreement with our previous studies^25–27^, Transactive Response DNA-binding protein-43 (TDP-43) mutants were supplied with *Mpe* or *Wse* at 0.1% w/w concentrations as adults (L-/A^+^), that, according to the life span curves, showed a lack of toxic effects (Supplementary Fig. S4). It is to be pointed out that we tried a higher concentration (1% w/w) and did not see any toxic effect. Even if it was not the aim of this study, at the concentration reported in the Supplementary Fig. S4, we observed a significant amelioration of the life span after *Mpe* and *Wse* treatment.

### Climbing assay

The climbing assay (negative geotaxis assay) was performed as previously reported^25–27,43,44^ with some modifications. Briefly, based upon age and in order to verify any age-dependent impairment, flies were divided into two groups (I: 3–6; II: 10–15 days old) of untreated-GAL4, *Mpe*-treated, and *Wse*-treated TDP-43 mutants. Cohorts of at least 60 flies from each experimental group were subjected to the assay. Flies were placed individually in a vertically-positioned plastic tube (length 10 cm; diameter 1.5 cm) and tapped to the bottom. Climbing time (seconds) was recorded upon crossing a line drawn at 6 cm from the bottom. Data were expressed as average ± standard error of the mean (SEM) from three to five experiment replications (n flies from 180 to 400). Statistically significant differences (p < 0.05) were analyzed between GAL4 vs. TDP-43 and between untreated-TDP-43 vs. treated ones by means of the one-way ANOVA (non parametric), followed by Dunn’s multiple comparisons test.

### Locomotor and sleep assay

The effects of *Mpe* and *Wse* were assayed at different age steps (at 7, 14 days treatment, hereafter referred to as group I and group II, respectively) in male TDP-43 e GAL4. Twenty - four (12 light:12 dark, thereafter indicated to as daytime and nighttime, respectively) hours of motor activity were recorded by *Drosophila* Activity Monitor System (DAMS – Trikinetics, Waltham, MA) from enclosure to 1 and 2 weeks of treatment with *Mpe* and *Wse* at 0.1% w/w. After one week and two weeks of treatment both mutants and GAL4 flies were isolated and each fly was housed in glass tubes (5 mm diameter by 65 mm length). Flies were given 24 hr to habituate to the experimental conditions prior to data collection. 10–15 flies were gathered and placed in each monitor for each genotype for each experiment. Motor activity in the tube was recorded and determined as each fly moves back and forth in the tube interrupting the infrared light beam that bisects each tube. Every time the fly crosses the tube is counted as a movement counted over 1-min periods. *Drosophila* Activity Monitor System was housed inside an environmental chamber where temperature and humidity were kept constant. Data for sleep analysis were acquired and analyzed with a dedicated software (pySolo)^45^. Sleep was defined as any period of uninterrupted behavioural immobility (0 counts/min) lasting >5 min according to Bushey *et al*.^32^. We considered, for each strain and age group, the total sleep, the number of episodes and the average of longest (AvLo) sleep episodes.

Statistically significant differences (p < 0.05) were analyzed between GAL4 vs. TDP-43 (*) and between untreated-TDP-43 vs. treated ones (#) by means of one-way ANOVA followed by HSD post-hoc test.

### Electrophysiological recordings

Flies from the different experimental groups were anesthetized by using CO_2_ and tightly anchored to a wax support with ventral side down, before starting the experiment, as previously reported^26^, and visualized under a stereomicroscope. Giant fiber system (GFS) was activated by a two tungsten stimulating electrodes, connected to a stimulator (Master 8, A.M.P.I, Jerusalem, IL, USA) triggered by a stimulus isolation unit (DS2A, Digitimer Ltd., Hertfordshire UK), were placed into both eyes of the fly. The intensity of each stimulus was not greater than 10 V and was increased gradually until the postsynaptic potential response was observed. Moreover, no correlation between stimulation intensity needed to evoke the response and type of animal tested was observed. As potential reference electrode, a ground tungsten wire was placed into the fly abdomen. A borosilicate recording electrode, shaped by a horizontal puller (P97, Flaming/Brown, Sutter Instruments, Novato, CA, USA) with a resistance of 4–5 MΩ when filled with 3 M KCl, was placed into the right or left backside of the fly along the 45a and 45b fibres of the dorsal longitudinal muscle fibres (DLMs). Evoked post-synaptic potentials (ePSPs) were recorded with an Axopatch 2-B amplifier (Axon Instruments, Foster City, CA), filtered at 0.5 kHz and digitized at 1 kHz. ePSPs were recorded in bridge mode, measured using peak and event detection software pCLAMP 8.2 (Axon Instruments, Foster City, CA), and analyzed off-line by pCLAMP 8.2 fit software (Axon Instruments, Foster City, CA). Experiments were blind to the treatment. Electrophysiological experiments were performed by applying a protocol consisting in a single GFS stimulation, delivered every 20 s, followed by the ePSP recording. The “frequency of following” was determined by delivering trains of 10 stimuli at increasing frequencies (from 10 to 200 Hz) indicating the failures, as the percentage of lacking responses at each train. As previously published^26,27,29^, we also evaluated the amplitude (peak of the PSP expressed in mV) as well as the latency (interval between the stimulating artifact and the time at ePSP peak, expressed in ms). In a separate set of flies, the recording electrode was placed in DLMs and events derived from spontaneous muscle contraction, and thus wing beats, were recorded in the absence or after 30 s of ether or chloroform vapour administration to the fly in order to induce a deep anaesthesia. After one minute of spontaneous recording, anaesthetic vapours were applied through a silicon tube placed close to the fly and recording has been continued for at least 5 min. This protocol was applied to all different experimental groups. Data are expressed as mean + SEM and analyzed by one or two-way ANOVAs followed by Bonferroni’s post-hoc tests.

## Electronic supplementary material

Video S1

Video S2

Video S3

Video S4

Video S5

Video S6

Supplementary information
