# Supplementary material for: Standardized phytotherapic extracts rescue anomalous locomotion and electrophysiological responses of TDP-43 Drosophila melanogaster model of ALS
Source: Sci Rep. 2018 Oct 30;8:16002. doi: 10.1038/s41598-018-34452-1 (PMC6207707; doi:10.1038/s41598-018-34452-1)
Supplement: Supplementary file 7 — Supplementary information [file 41598_2018_34452_MOESM7_ESM.pdf]

Supplementary information

Standardized phytotherapic extracts rescue anomalous locomotion and electrophysiological responses of TDP-43 *Drosophila melanogaster* model of ALS

Riccardo Maccioni[1+], Maria Dolores Setzu[1+], Giuseppe Talani[2+], Paolo Solari[1], Ameya Kasture[3], Sonja Sucic[3], Simona Porru[4], Patrizia Muroni[1], Enrico Sanna[4], Sanjay Kasture[5], Elio Acquas[4], Anna Liscia[1]

[1]University of Cagliari, Department of Biomedical Sciences, Monserrato, 09042, Italy
[2]National Research Council (CNR), Institute of Neuroscience, Monserrato, 09042, Italy
[3]Medical University of Vienna, Institute of Pharmacology, Center of Physiology and Pharmacology, Vienna, A-1090, Austria
[4]University of Cagliari, Department of Life and Environmental Sciences, Cagliari, 09126, Italy
[5]Pinnacle Biomedical Research Institute, Bhopal, 462003, India.

+these authors contributed equally to this work
*liscia@unica.it

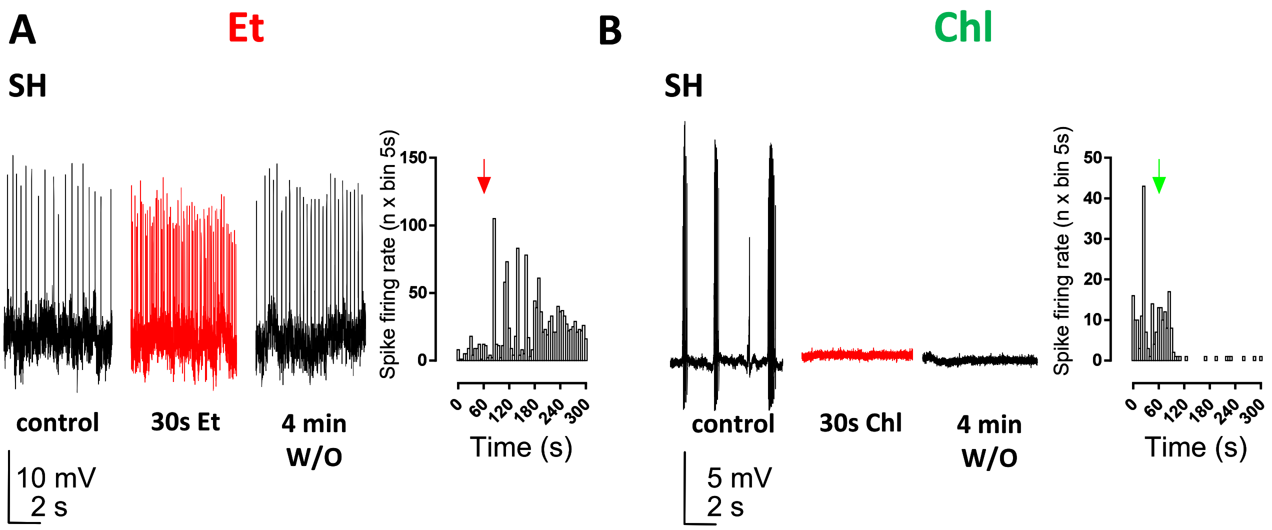

Supplementary Figure S1: Representative traces and plots obtained from SH mutant flies, in which spontaneous PSPs were recorded in the absence, after 30 s and after 4 min of ether (Et) or chloroform (Chl) exposure. Arrows in graphs indicate when vapours were delivered. Scale bars: left panels, 10 mV/2 s, right panels 5 mV/2 s.

natural REMEDIES
QUALITY CONTROL DEPARTMENT

TLC PROFILE

**Product Name**	**:**	***Mucuna pruriens* extract (≥ 10% L-Dopa)**
**Batch No.**	**:**	**RD/3208**
**Reference extract used for comparison**		**MP/140101**

254nm

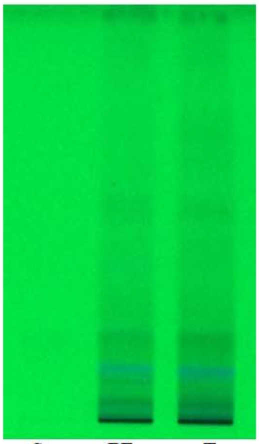

S RE T

366nm

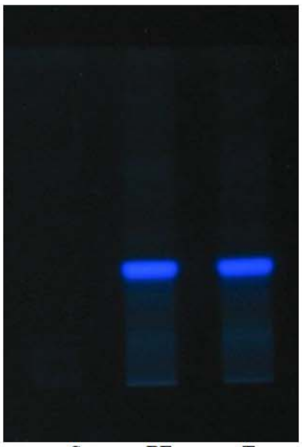

S RE T

Visible light after derivatization

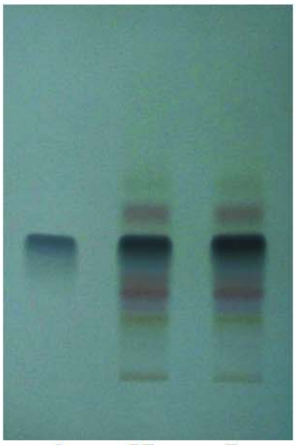

S RE T

**S**	**:**	**L-Dopa reference standard**	**B. No. 59200**
**RE**	**:**	***Mucuna pruriens* reference extract (prepared using crude drug reference standard (CDRS) B. No. 386)**	**B. No. MP/140101**
**T**	**:**	***Mucuna pruriens* extract**	**B. No. RD/3208**

**METHOD ADOPTED**		
**SOP No.**	**:**	NR/QCD/APM05 WI(31)
**Mobile Phase**	**:**	Butanol : Ethanol : Acetic acid : Water (60 : 32 : 12: 8)
**Detection**	**:**	Ninhydrine (30mg of ninhydrine in 10ml of Butanol & 0.3ml of Glacial acetic acid)
**Standard Preparation**	**:**	5 mg of L-Dopa dissolved in 5ml of 30%v/v aqueous Orthophosphoric acid solution diluted with methanol to 10ml.
**Sample Preparation**	**:**	500mg of extract + 10ml 30%v/v aqueous Orthophosphoric acid solution diluted to 100ml with methanol.

Natural Remedies Private Limited
Regd. Office & R&D
CIN No: U24232KA1998PTC023573
5B, Veersandra Industrial Area, Hosur Road, Electronic City Phase 2, Bangalore 560100. Karnataka – INDIA
Tel: 91 8040209999/8/7,27382265, Fax: 91 80 40209817, Web: www.naturalremedy.com
E-Mail: qc@naturalremedy.com, info@naturalremedy.com

Supplementary Figure S2: Details of the batch of *Mucuna pruriens* used in this study (Page 1).

QUALITY CONTROL DEPARTMENT

HPLC PROFILE

Reference standard chromatogram

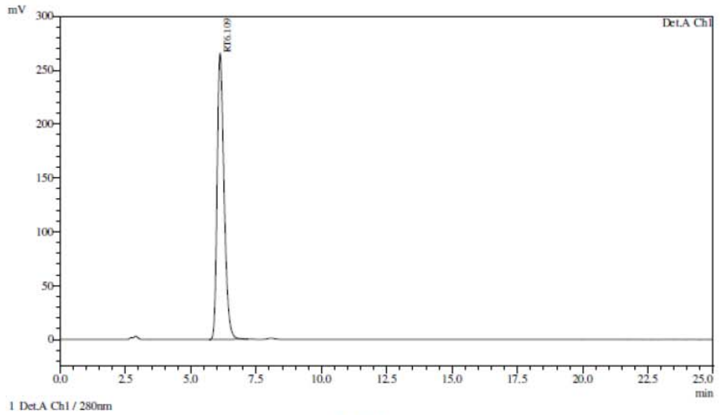

1 Det.A Ch1 / 280nm

PeakTable

Detector A Ch1 280nm

Peak#	Name	Ret. Time	Area	Height	Area %	Height %
1	RT6.109	6.109	4891567	265935	100.000	100.000
Total			4891567	265935	100.000	100.000

Extract chromatogram – *Mucuna pruriens* extract (B. No. RD/3208)

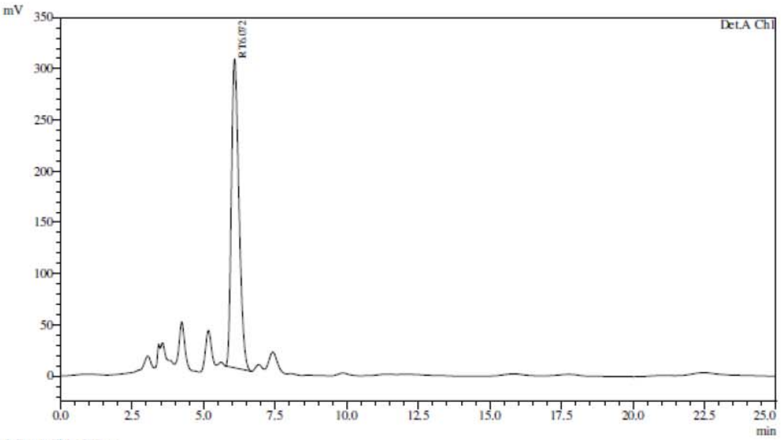

1 Det.A Ch1 / 280nm

PeakTable

Detector A Ch1 280nm

Peak#	Name	Ret. Time	Area	Height	Area %	Height %
1	RT6.072	6.072	5547471	301812	100.000	100.000
Total			5547471	301812	100.000	100.000

Natural Remedies Private Limited
Regd. Office & R&D
CIN No: U24232KA1998PTC023573
5B, Veersandra Industrial Area, Hosur Road, Electronic City Phase 2, Bangalore 560100. Karnataka – INDIA
Tel: 91 8040209999/8/7,27382265, Fax: 9180 40209817, Web: www.naturalremedy.com
E-Mail: qc@naturalremedy.com, info@naturalremedy.com

Supplementary Figure S2: Details of the batch of *Mucuna pruriens* used in this study (Page 2).

QUALITY CONTROL DEPARTMENT

METHOD ADOPTED		
**SOP No.**	:	NR/QCD/APM06 WI(31)
**Mobile phase**	:	Buffer : Acetonitrile<br>92 : 8<br>(Buffer: 1.36gm of potassium di hydrogen orthophosphate to 900ml of water and adjust the pH to 2.5 using orthophosphoric acid).
**Column**	:	C18-ODS. 5µ size, 250 x 4.6 mm (Merck).
**Flow Rate**	:	2.0 ml/ min
**Wave Length**	:	280 nm
**Standard Preparation**	:	500mcg/ml L-Dopa is dissolved in 5ml 30% orthophosphoric acid. Add water and make up to 100ml.
**Sample Preparation**	:	5000mcg/ml of extract is dissolved in 5 ml of 30% orthophosphoric acid. Add water and make up to 100ml.

Natural Remedies Private Limited
Regd. Office & R&D
CIN No: U24232KA1998PTC023573
5B, Veersandra Industrial Area, Hosur Road, Electronic City Phase 2, Bangalore 560100. Karnataka – INDIA
Tel: 91 8040209999/8/7,27382265, Fax: 91 80 40209817, Web: www.naturalremedy.com
E-Mail: qc@naturalremedy.com, info@naturalremedy.com

Supplementary Figure S2: Details of the batch of *Mucuna pruriens* used in this study (Page 3).

natural™
REMEDIES
QUALITY CONTROL DEPARTMENT

TLC PROFILE

**Product Name**	**:**	***Withania somnifera* extract**
**Batch No.**	**:**	**PC/FWS1701003**
**Reference extract used for comparison**	**:**	**WS/170101**
**Source of reference standard**	**:**	**Natural Remedies Pvt. Ltd.**

254nm **366nm** **Visible light after derivatisation**

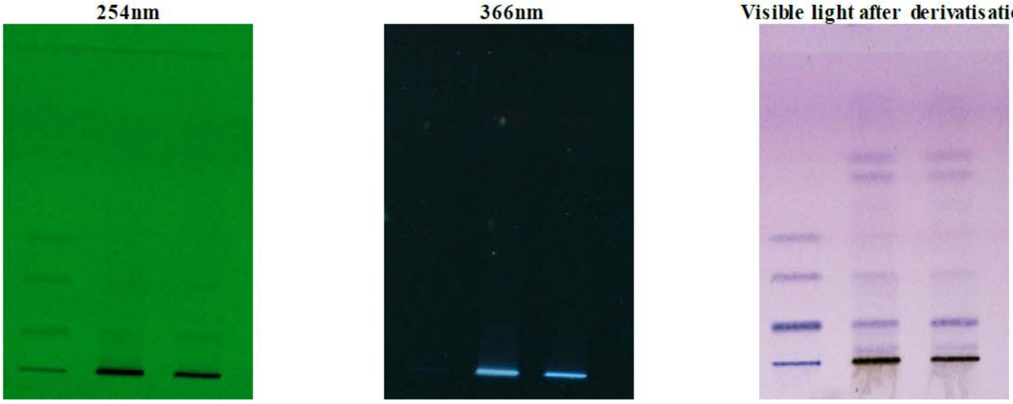

S1 T1 T2 **S1 T1 T2** **S1 T1 T2**

S1	:	Withaferin-A reference standard	B. No. T12F047
		Withanolide-A reference standard	B. No. T13B057
		Withanolide-B reference standard	B. No. T13K088
		Withanoside-IV reference standard	B. No. T13B107
		Withanoside-V reference standard	B. No. T14D096
		12 Deoxy withastramonolide reference standard	B. No. T14A099
T1	:	*Withania somnifera* reference extract (prepared using Crude drug reference standard (CDRS) B. No. RHD-115)	B. No. WS/170101
T2	:	*Withania somnifera* extract	B. No. PC/FWS1701003

**METHOD ADOPTED**		
**SOP No.**	**:**	NR/QCD/APM05 WI(17)
**Mobile Phase**	**:**	Ethyl Acetate : Toluene : Acetic acid<br>45 : 55 : 3
**Detection**	**:**	Anisaldehyde Sulphuric acid
**Standard Preparation (All withanolides)**	**:**	0.5mg of each withanolides per ml in Methanol
**Sample Preparation**	**:**	3g of extract in 50ml Methanol

Natural Remedies Private Limited
Regd. Office & R&D
CIN No: U24232KA1998PTC023573
5B, Veersandra Industrial Area, Hosur Road, Electronic City Phase 2, Bangalore 560100. Karnataka – INDIA
Tel: 91 8040209999/8/7,27382265, Fax: 9180 40209817, Web: www.naturalremedy.com
E-Mail: qc@naturalremedy.com, info@naturalremedy.com

Supplementary Figure S3: Details of the batch of *Withania somnifera* used in this study (Page 1).

natural™
REMEDIES
QUALITY CONTROL DEPARTMENT

HPLC PROFILE

Chromatogram - Standard mix Withaferin-A (B. No. T12F047), Withanolide-A (B. No. T13B057), Withanolide-B (B. No. T13K088), Withanoside IV (B. No. T13B107), Withanoside V (B. No. T14D096), 12-Deoxywithastramonolide reference standard (B. No. T14A099)

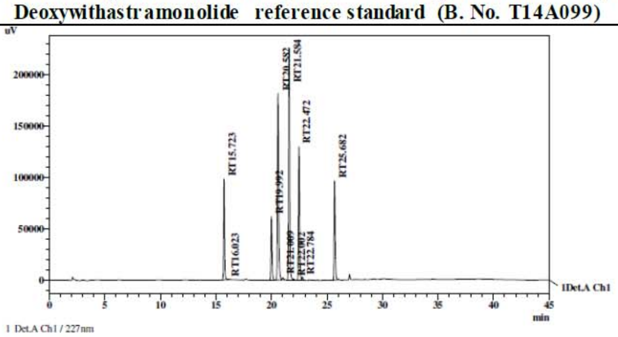

PeakTable @E:\2017\Glycyrrhiza\JAn 2017\17.01.2017\Data004.lcd

Detector A Ch1 227nm

Peak#	Name	Ret. Time	Area	Area %
1	RT15.723	15.723	597483	11.102
2	RT16.023	16.023	3585	0.067
3	RT19.992	19.992	425917	7.914
4	RT20.582	20.582	1324782	24.616
5	RT21.009	21.009	17610	0.327
6	RT21.584	21.584	1505834	27.980
7	RT22.002	22.002	9026	0.168
8	RT22.472	22.472	855200	15.890
9	RT22.784	22.784	19897	0.370
10	RT25.682	25.682	622501	11.567
Total			5381834	100.000

Reference Extract chromatogram – *Withania somnifera* extract (B. No WS/170101)

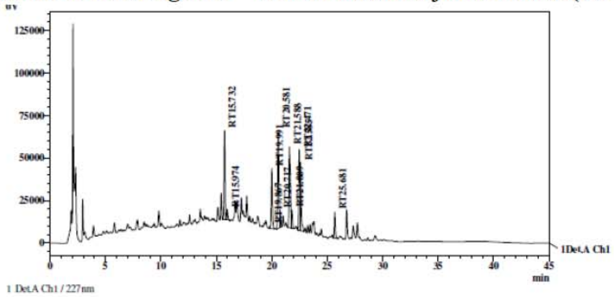

PeakTable @E:\2017\Glycyrrhiza\JAn 2017\17.01.2017\Data006.lcd

Detector A Ch1 227nm

Peak#	Name	Ret. Time	Area	Area %
1	RT15.732	15.732	310879	13.994
2	RT15.974	15.974	39230	1.766
3	RT19.867	19.867	5125	0.231
4	RT19.991	19.991	245942	11.071
5	RT20.581	20.581	409007	18.411
6	RT20.717	20.717	51603	2.323
7	RT21.588	21.588	442010	19.896
8	RT21.809	21.809	65554	2.951
9	RT22.471	22.471	278104	12.518
10	RT22.585	22.585	275250	12.390
11	RT25.681	25.681	98850	4.450
Total			2221554	100.000

Natural Remedies Private Limited
Regd. Office & R&D
CIN No: U24232KA1998PTC023573
5B, Veersandra Industrial Area, Hosur Road, Electronic City Phase 2, Bangalore 560100. Karnataka – INDIA
Tel: 91 8040209999/8/7,27382265, Fax: 91 80 40209817, Web: www.naturalremedy.com
E-Mail: qc@naturalremedy.com, info@naturalremedy.com

Supplementary Figure S3: Details of the batch of *Withania somnifera* used in this study (Page 2).

QUALITY CONTROL DEPARTMENT

Extract chromatogram – *Withania somnifera* extract (Batch No. PC/FWS1701003)

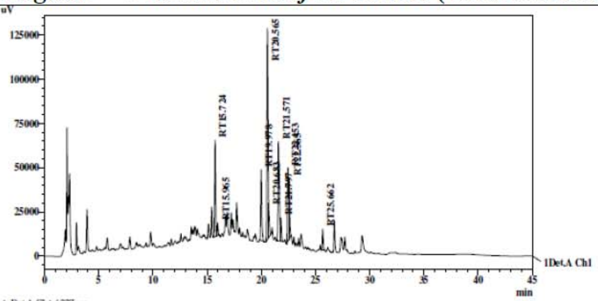

1 Det.A Ch1 / 227nm

PeakTable @E:\2017\Withania\Jan 2017\17.01.2017\Data007.lcd

Detector A Ch1 227nm

Peak#	Name	Ret. Time	Area	Area %
1	RT15.724	15.724	321897	12.139
2	RT15.965	15.965	41570	1.568
3	RT19.978	19.978	285208	10.755
4	RT20.565	20.565	818719	30.874
5	RT20.683	20.683	73300	2.764
6	RT21.571	21.571	509448	19.211
7	RT21.797	21.797	86327	3.255
8	RT22.453	22.453	255179	9.623
9	RT22.565	22.565	189145	7.133
10	RT25.662	25.662	71019	2.678
Total			2651810	100.000

METHOD ADOPTED		
**SOP No.**	:	NR/QCD/APM04 WI(17)
**Mobile phase**	:	1) Buffer: 0.01N potassium dihydrogen phosphate in water and 0.5ml orthophosphoric acid.<br>2) Acetonitrile (Gradient Elution)
**Column**	:	LichroCART, Lichrospher, C18-5µ (Merck)
**Flow Rate**	:	1.500ml/mi
**Wave Length**	:	227nm
**Standard Preparation (All withanolides)**	:	0.5mg of each withanolides per ml in Methanol
**Sample Preparation**	:	40mg/ml of extract in Methanol

Natural Remedies Private Limited
Regd. Office & R&D
CIN No: U24232KA1998PTC023573
5B, Veersandra Industrial Area, Hosur Road, Electronic City Phase 2, Bangalore 560100. Karnataka – INDIA
Tel: 91 8040209999/8/7,27382265, Fax: 91 80 40209817, Web: www.naturalremedy.com
E-Mail: qc@naturalremedy.com, info@naturalremedy.com

Supplementary Figure S3: Details of the batch of *Withania somnifera* used in this study (Page 3).

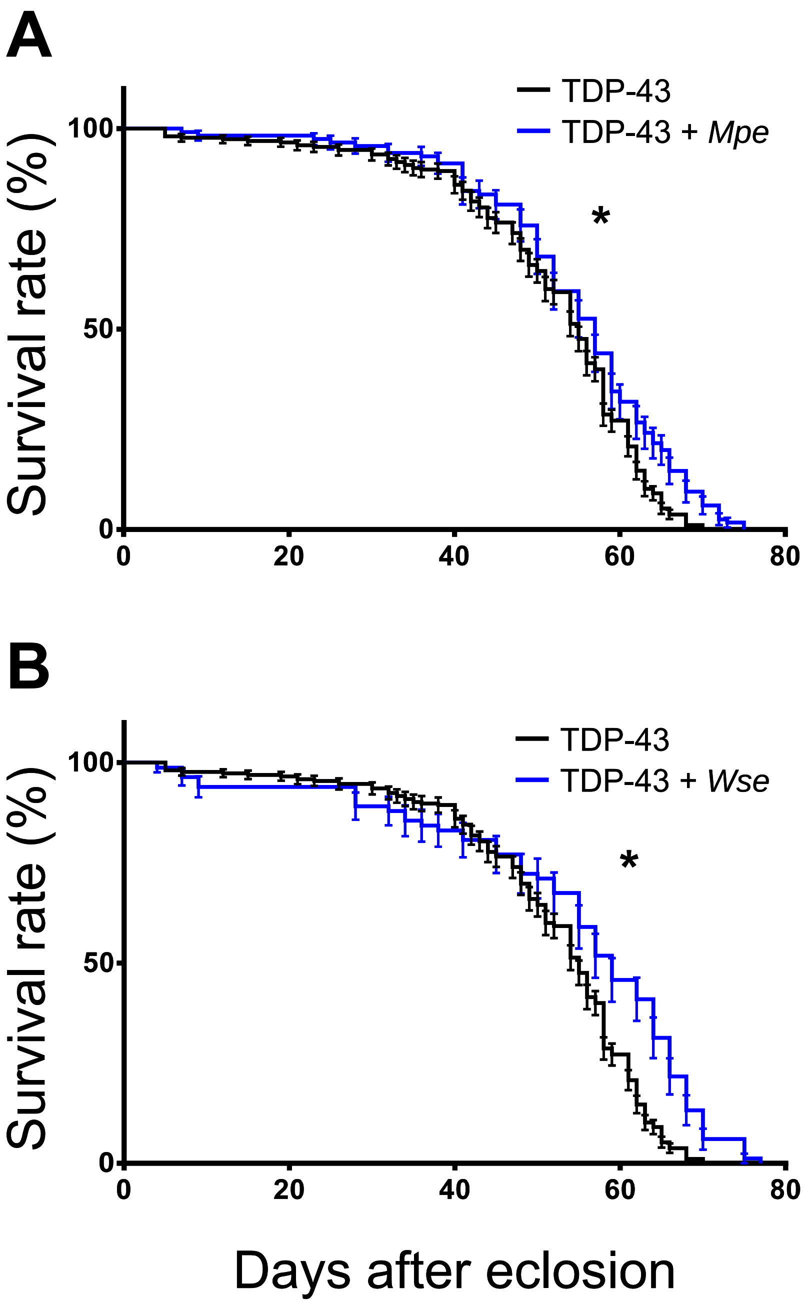

Supplementary Figure S4: Effects of *Mucuna pruriens* extract (*Mpe*) and *Withania somnifera* extract (*Wse*) treatment on lifespan of TDP-43 mutant. * Indicates $p < 0.05$ at Kaplan-Meier survival curves (Gehan-Breslow-Wilcoxon-Graph Pad Prism 5.01).

Supplementary Video S1: *Drosophila* TDP-43 mutants showing spastic paralysis coupled to shaker-like movements after ether supply.

Supplementary Video S2: *Drosophila* TDP-43 mutants showing spastic paralysis coupled to shaker-like movements after chloroform supply.

Supplementary Video S3: *Drosophila* GAL4 flies do not show any particular movement after ether supply.

Supplementary Video S4: *Drosophila* SH flies do not show any particular movement after chloroform supply.

Supplementary Video S5: *Drosophila Wse*-treated TDP-43: treatment prevents the effect of ether administration observed in untreated TDP-43 mutant flies.

Supplementary Video S6: *Drosophila Mpe*-treated TDP-43: treatment prevents the effect of ether administration observed in untreated TDP-43 mutant flies.
